# The prevalence of freezing of gait in Parkinson’s disease and in patients with different disease durations and severities

**DOI:** 10.1186/s41016-020-00197-y

**Published:** 2020-05-14

**Authors:** Hong-Liang Ge, Xiao-Yong Chen, Yuan-Xiang Lin, Ting-Juan Ge, Liang-Hong Yu, Zhang-Ya Lin, Xi-Yue Wu, De-Zhi Kang, Chen-Yu Ding

**Affiliations:** 1 Department of Neurosurgery, The First Affiliated Hospital of Fujian Medical University, No. 20 Chazhong Road, Fuzhou, 350004 Fujian Province People’s Republic of China; 2 Brain Center, Quzhou Second People Hospital, Quzhou, People’s Republic of China

**Keywords:** Prevalence, Freezing, Gait, Parkinson disease

## Abstract

**Background:**

The prevalence rates of freezing of gait (FOG) in Parkinson’s disease (PD) vary widely, ranging from 14.0 to 55.1%. Our aim is to calculate the overall prevalence of FOG in all PD patients with different disease durations and severities.

**Methods:**

Using Medline/PubMed/Embase, we carried out a systematic literature search for studies reporting the PD and clinically relevant FOG.

**Results:**

After primary screening, a total of 35 studies were identified and further analyzed for inclusion into the analysis, and 29 studies fulfilled the quality criteria and included in this meta-analysis. The overall prevalence of FOG in PD was 39.9% (95% CI 35.3-44.5%). The FOG identified by the freezing of gait questionnaire item 3 may be more prevalent (43.8%, 95% CI 38.5-49.1%) than the FOG identified by the Unified Parkinson’s Disease Rating Scale item 14 (36.0%, 95% CI 29.0-43.1%). Disease duration and severity are both the clinical features associated with the FOG. The highest FOG prevalence rate in PD patients was seen in patients with disease durations ≥ 10 years, at 70.8%, followed that of PD patients with disease durations ≥ 5 years (53.3%), and PD patients with disease durations < 5 years (22.4%). FOG presented in 28.4% of PD patients with Hoehn and Yahr staging (H&Y) score ≤ 2.5, and in 68.4% of PD patients with H&Y score ≥ 2.5.

**Conclusion:**

This meta-analysis confirms that the prevalence of FOG in PD is considerable, and highlights the need for accurate identification of FOG in PD.

## Background

Parkinson’s disease (PD) is a neurodegenerative disease primarily characterized by rigidity, bradykinesia, and resting tremor; however, the freezing of gait (FOG) is also a common and disabling symptom in PD [1–3]. FOG is characterized by sudden and brief episodes of inability to produce effective forward stepping [4–6]. Therefore, FOG proposes major risks for falls, and leading to disability to patients, making the efficient identification of it important [7].

Although FOG is common in PD, it has not been extensively studied. The prevalence of FOG in PD patients that are reported in the literature vary widely, ranging from 14.0 to 55.1% [8, 9]. Up to 86.5% of advanced PD patients experience FOG [10], and up to 37.8% of early PD patients have the FOG as defined by a validated scale [11]. The different rates reported could be caused by the way the FOG is diagnosed, the nature of the PD patients being studied, the date of study conducted, the quality of studies, and the geographical region. Given this background, the present meta-analysis aims to calculate the overall prevalence of FOG in all PD patients, and to calculate the FOG prevalences in PD patients with different disease durations and severities.

## Methods

### Search strategy

This meta-analysis was performed according to PRISMA guideline [12]. A systematic literature search was carried out using databases Medline/PubMed/Embase. The entire time scale was used up to April 23, 2019. To include all actual literature on FOG in PD, we used the following terms: “freezing” or “gait” or “frozen” or “FOG” in combination with “Parkinson”. Pubmed was searched by text keywords and Medical Subject Heading terms. Embase was searched by Emtree and text words. Our results were limited to the humans and English language literature.

### Quality assessment

The reliability of the literature was assessed with the modified quality assessment of diagnostic accuracy studies (QUADAS) tool, which is an efficient quality assessment system [13, 14] and was previously used to determine the prevalence of anxiety [15] and pain [16] in PD. Ten independent scores are included in the criteria used to evaluate the quality of the included studies using the modified QUADAS tool (Table 1). The score ranges from 0 to 19 points, with > 13 points (corresponds to 75% of the highest score) being the cut-off level of methodological acceptability [15, 16]. The studies were independently reviewed by 2 authors (HLG and XYC). In case of discrepancies between the raters, a decision was made after reassessment by the corresponding authors (CYD).

**Table 1:** Modified quality assessment of diagnostic accuracy studies tool: quality criteria for prevalence studies

Sorts	Subsorts	Quality criteria
A		The final sample should be representative of the target population
	1	At least 1 of the following should apply for the study (2 points)
		– An entire target population
		– Randomly selected sample
		– Sample stated to represent the target population
	2	At least one of the following (2 points)
		– Reasons for nonresponders described
		– Nonresponders described
		– Comparison of responders and nonresponders
		– Comparison of sample and target population
	3	Response rate > 90% (2 points)
		Response rate 70% to 90% (1 point)
		Response rate ≤ 70% (0 points)
B		Quality of data
	4	Were the data primary from a prevalence study (2 points) or was it taken from a survey not specifically designed for that purpose (1 point)?
	5	The same mode of data collection should be used for all subjects (2 points), if not: 1 point
	6	– The data have been collected directly from the patient by means of a validated questionnaire/interview (3 points)
		– No validated questionnaire/interview patients (2 points)
		– Data have been collected from proxies or retrospectively from medical record (1 point).
C		General description of the method and results should include:
	7	Description of target population and setting where patients were found (2 points)
	8	Description of stage of disease, sex, age (all 2 points, 1 or 2: 1 point)
	9	Final sample size (1 point)
D		Definition of FOG prevalence
	10	Prevalence recall periods should be stated (1 point)

### Data extraction and validity assessment

Two authors (HLG and XYC) independently reviewed the full texts of the selected studies. Any disagreement between the two reviewers was resolved by the verdict of the corresponding authors (CYD). The interested variables included the clinical settings, prevalence rates of FOG, sample sizes, the scale used to diagnose the FOG, disease durations, and disease severities. The disease severity was measured by the Hoehn and Yahr staging (H&Y) scale. Higher score in H&Y corresponds to increased severity [17, 18]. The diagnostic criteria of FOG varied across studies. Authors of different studies chose the diagnostic criteria of FOG based on their own experiences. For this analysis, we followed the criteria stated by the authors of the selected studies.

### Heterogeneity and statistical analyses

The statistical heterogeneity was calculated using the chi-square (Chi2) test at 10% significant level. If the value was > 50%, a random-effect model was used. Otherwise, a fixed-effect model was adopted. The random-effects model should be used because these studies had a high heterogeneity [19, 20]. Sub-group analysis and visual inspection of the data were performed to further investigate potential sources of heterogeneity. Chi-square test and hypothesis testing were used to compare the prevalence of objectively diagnosed FOG with subjectively diagnosed one. Considering the different statistical weights of the paper selected, we conducted a forest plot to solve this problem.

Meta-analysis was executed with STATA (version 12.0, Stata Corporation, College Station, TX, USA) to obtain the overall prevalence of FOG in PD. After estimating the overall FOG prevalence in PD patients, articles were first assessed for FOG prevalence based on the different diagnostic criteria then in PD patients with different disease duration, and lastly in PD patients with different disease severities.

## Results

### Description of included studies

**Fig. 1:**
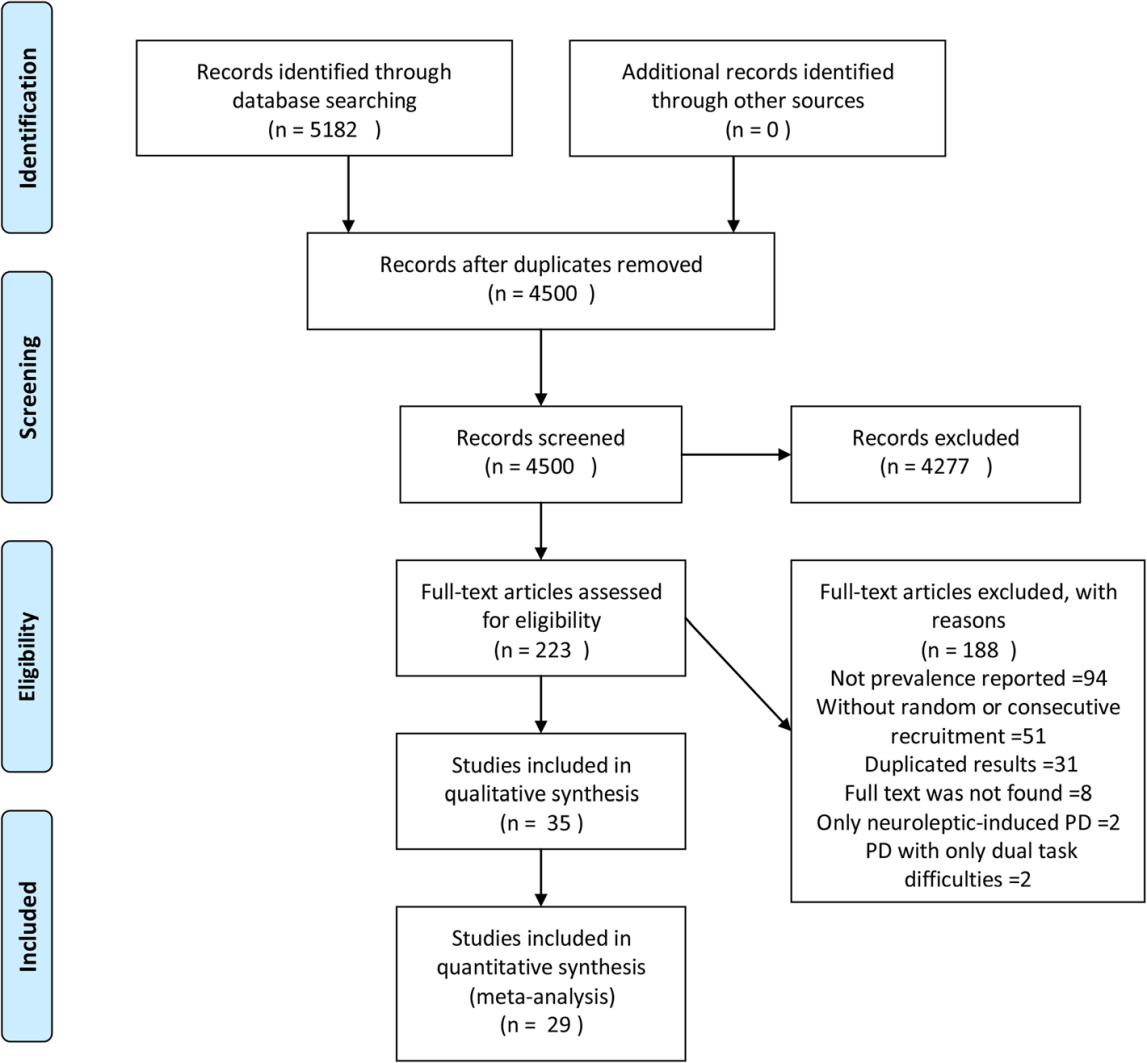
PRISMA 2009 flow diagram

We used the following terms: “freezing” or “gait” or “frozen” or “FOG” in combination with “Parkinson”, which revealed 4500 articles after removing duplicates. Subsequently, the abstracts of these articles were read, reviews, and studies with only deep brain stimulation patients and unclear criteria for the diagnosis of FOG were excluded. All potential studies reporting the prevalence of FOG in PD patients were then read in full for eligibility and 188 articles were excluded for the following reasons: prevalence of FOG in PD was not mentioned (*n* = 94), there may be an insufficient description of a random or consecutive design of patient recruitment (*n* = 51), the publications stemmed from the same database or duplicate articles (*n* = 31), the population included had only neuroleptic-induced PD or PD with only dual-task difficulties (*n* = 4), or the full text was not found (*n* = 8). The remaining 35 studies were included in the qualitative analysis (Fig. 1).

Of the total of 35 studies included in the qualitative analyses, 16 focused on the FOG as a primary objective and the remaining studies reported FOG as a secondary outcome. Twenty-nine studies (82.9%) were further analyzed as they met the cut-off score of 14 points on the QUADAS tool [8–11, 21–46]. All included studies were conducted after the year 2000, with approximately 72.4% of the articles published in the past 5 years, which likely reflects the increasing awareness in the past decade of FOG in PD.

Of the 29 studies meeting the quality criteria, 11 studies included patients from Europe, 8 from the USA, 4from Australia, 2 from China, and 4 from Israel. The overview of the studies included in this meta-analysis is illustrated in Table 2, and the prevalence of FOG reported in these studies is shown in Table 3.

**Table 2:** Overview of the studies included in this meta-analysis

Study	Country	Sample size	Quality score	Identification of FOG	Age (years)	Disease duration (years)	H&Y
Margolesky et al [36]	United States	102	19	freezing of gait was self-reported	68.0 ± 10.0	5.3 ± 4.8	1-4
Mckay et al [46]	United States	65	18	FOG-Q item 3 > 1 or UPDRS item 14 > 1	68 ± 10	7.3 ± 5.6	1-3
Mancini et al [40]	Israel	94	16	new FOG-Q part I = freezer	65.4 ± 9.7 for non-freezers; 64.2 ± 8.8 for freezers	4.9 ± 2.8 for non-freezers; 7.6 ± 4.4 for freezers	2.4 ± 0.5 for non-freezers; 3.2 ± 0.8 for
Djaldetti et al [37]	Israel	41	14	UPDRS item 14 > 1	63.5 ± 9.9	9.5 ± 3.2	1-3
Ehgoetz Martens et al [38]	Australia	221	15	FOG-Q item 3 ≥ 1	65.4 ± 9.8 for non-freezers; 70.8 ± 9.4 for transitional freezers; 70.2 ± 10.9 for continuing freezers	3.2 ± 4.3 for non-freezers; 6.0 ± 4.1 for transitional freezers; 9.7 ± 7.5 for continuing freezers	ND
Kader et al [44]	Sweden	243	16	FOG-Q item 3 ≥ 1	70 ± 9.2	8 (1–43)^a^	3 (2–3) ^a^
Sutter et al [45]	United States	111	16	new FOG-Q item 1 > 0	66.5 ± 1.7	4.0 (2.0-8.0)^a^	1-4
Forsyth et al [43]	Australia	82	16	new FOG-Q	66.5 ± 7.6	7.5 ± 5.7	2.0 ± 0.7
Ricciardi et al [41]	Italy	43	15	new FOG-Q item 1 > 0	68.0 ± 11.6	5.3 ± 5.4 for non-freezers; 9.5 ± 5.8 for freezers	ND
Vervoort et al [42]	Belgium	76	16	new FOG-Q or FOG occurrence in the lab	60.6 ± 10.0	6.8 ± 4.6	2.1 ± 0.5
Shin et al [35]	United states	141	14	freezing of gait was self-reported	69.7 ± 8.21	6.34 ± 4.84	ND
Allen et al [21]	Australia	231	14	freezing of gait was self-reported	70.6 ± 8.8	7.9 ± 5.9	ND
Amboni et al [11]	Italy	593	19	FOG-Q item 3 ≥ 1	66.1 ± 9.7 for non-freezers; 67.5 ± 8.7 for freezers	6.1 ± 3.8 for non-freezers; 9.8 ± 4.8 for freezers	1-5
Forsaa et al [22]	Norway	232	17	UPDRS item 14 ≥ 1	64.9 (28.2-86.0) ^b^	8.6 ± 5.7	1-5
Gazibara et al [23]	Serbia	300	17	new FOG-Q item 1 > 0	61.4 (22–83)^b^	0.5-30	1-4
Lieberman et al [24]	United States	212	14	UPDRS item 14 ≥ 2	ND	All patients < 5	1-5
Lindholm et al [25]	Sweden	141	15	FOG-Q item 3 ≥ 1	68 ± 9.7	4 ± 3.9	1-5
Walton et al [26]	Australia	203	15	FOG-Q item 3 ≥ 1	66.77 ± 8.9	5.1 ± 5.1	1-3
Bohnen et al [8]	United States	143	14	MDS-UPDRS item 3.11 ≥ 1	65.5 ± 7.4	6.0 ± 4.3	2.4 ± 0.5
Ou et al [27]	China	474	16	FOG-Q item 3 ≥ 1	62.09 ± 10.55	4.77 ± 4.04	1-5
Perez-Lloret et al [28]	French	672	19	UPDRS item 14 ≥ 1	50.7% patients ≥68	50.7% patients > 5	1-5
Auyeung et al [10]	China	171	17	sudden and transient blocks of movement while walking	62.2 ± 10.6	All patients > 10	1.5-5
Contreras et al [29]	Spain	160	16	FOG-Q item 3 ≥ 1	72.0 ± 9.5	8.1 ± 6.4	2.6 ± 1.0
Garcia-Ruiz et al [30]	Spain	45	15	sudden and transient blocks of movement while walking	58.5 ± 10.2	a follow-up study of new-onset PD	
Josiah et al [31]	United States	916	14	UPDRS item 14 ≥ 1	67.1 ± 11.0	6.6 ± 6.0	ND
Coelho et al [32]	Spain and Portugal	50	15	ND	74.1 ± 7.0	17.9 ± 6.3	4-5
Moore et al [9]	Israel	118	16	FOG-Q item 3 ≥ 1	65.8 ± 10.2	8.5 ± 5.8	2.7 ± 0.8
Giladi et al [33, 34]	United States	800	15	UPDRS item 14 ≥ 1	61.1 ± 9.5	2.1 ± 1.3	1-2
Giladi et al [33, 34]	Israel	172	16	feet get glued to the ground was self-reported	58.3 ± 13.2	11.8 ± 5.6	1-5

*H&Y* Hoehn and Yahr staging, *FOG-Q* freezing of gait questionnaire, *UPDRS* unified Parkinson’s disease rating scale, *MDS-UPDRS* movement disorder society revised Unified Parkinson’s Disease Rating Scale, *PD* Parkinson’s disease, *ND* no data

^a^median (q1-q3)

^b^mean (range)

**Table 3:** The prevalence of FOG in Parkinson’s disease reported in these literatures

Study	FOG, % ( *n* of freezer/*n* of PD patients)					
	In general PD patients	Duration < 5 years	Duration ≥ 5 years	Duration ≥ 10 years	H&Y ≤ 2.5	H&Y > 2.5
Margolesky et al [36]	26.5% (27/102)					
Mckay et al [46]	40.0% (26/65)^c^				33.3% (15/45)	55.0% (11/20)
Mancini et al [40]	26.6% (25/94)^a^					
Djaldetti et al [37]	34.8% (15/41)		34.8% (15/41)			
Ehgoetz Martens et al [38]	41.6% (92/221)					
Kader et al [44]	56.8% (138/243)^a^					
Sutter et al [45]	41.4% (46/111)^a^				37.0% (34/92)	63.2% (12/19)
Forsyth et al [43]	39.0% (30/82)^a^					
Ricciardi et al [41]	55.8% (24/43)*					
Vervoort et al [42]	22.4% (17/76)^a^					
Shin et al [35]	38.6% (54/141)	29.2% (21/73)	48.4% (31/64)	59.1% (13/22)		
Allen et al [21]	49.4% (114/231)					
Amboni et al [11]	54.8% (325/593)^a^	27.4% (48/175)	65.8% (271/412)	78.3% (148/189)	35.3% (124/351)	82.9% (194/234)
Forsaa et al [22]	27.2% (63/232) ^b^			62.5% (145/232)		
Gazibara et al [23]	36.7% (110/300)					
Lieberman et al [24]		12.7% (27/212)				
Lindholm et al [25]	41.1% (58/141)^a^					
Walton et al [26]	42.4% (86/203) ^a^					
Bohnen et al [8]	14.0% (20/143)					
Ou et al [27]	46.6% (221/474) ^a^	36.1% (108/299)	64.6% (113/175)		33.5% (115/343)	82.2% (106/129)
Perez-Lloret et al [28]	38.2% (257/672) ^b^	25.4% (85/335)	51.0% (172/337)		31.5% (174/553)	60.0% (150/250)
Auyeung et al [10]				86.5% (148/171)		
Contreras et al [29]	44.4% (71/160)^a^					
Garcia-Ruiz et al [30]		4.4% (2/45)	44.4% (20/45)	68.9% (31/45)		
Josiah et al [31]	41.5% (380/916)^b^					
Coelho et al [32]				62.0% (31/50)		62.0% (31/50)
Moore et al [9]	55.1% (65/118) ^a^					
Giladi et al (2001)					7.1% (57/800)	
Giladi et al (2001)			53.5% (92/172)		22.2% (10/45)	63.8% (81/127)

*FOG-Q* freezing of gait questionnaire, *UPDRS* unified Parkinson’s disease rating scale, *MDS-UPDRS* movement disorder society revised Unified Parkinson’s Disease Rating Scale, *FOG* freezing of gait, *PD* Parkinson’s disease, *H&Y* Hoehn and Yahr staging

^a^According to the FOG-Q and new FOG-Q

^b^According to UPDRS and MDS-UPDRS

^c^According to the FOG-Q or UPDRS

A total of 22 studies included both early and advanced PD was used to calculate the overall estimated mean prevalence of FOG in all PD patients. Of these 22 studies, 13 reported FOG according to the freezing of gait questionnaire item 3 (FOG-Q item 3); 3 reported FOG according to the Unified Parkinson’s Disease Rating Scale item 14 (UPDRS item 14); and 5 reported FOG according to other criteria (3 were self-reported); and 1 reported FOG according to FOG-Q item 3 or UPDRS item 14.

The remaining 7 studies included patients with the limitation in disease duration or disease severity. They were accepted in the supplemental analysis performed based on the disease durations and disease severities of PD patients.

### Prevalence of FOG

**Fig. 2:**
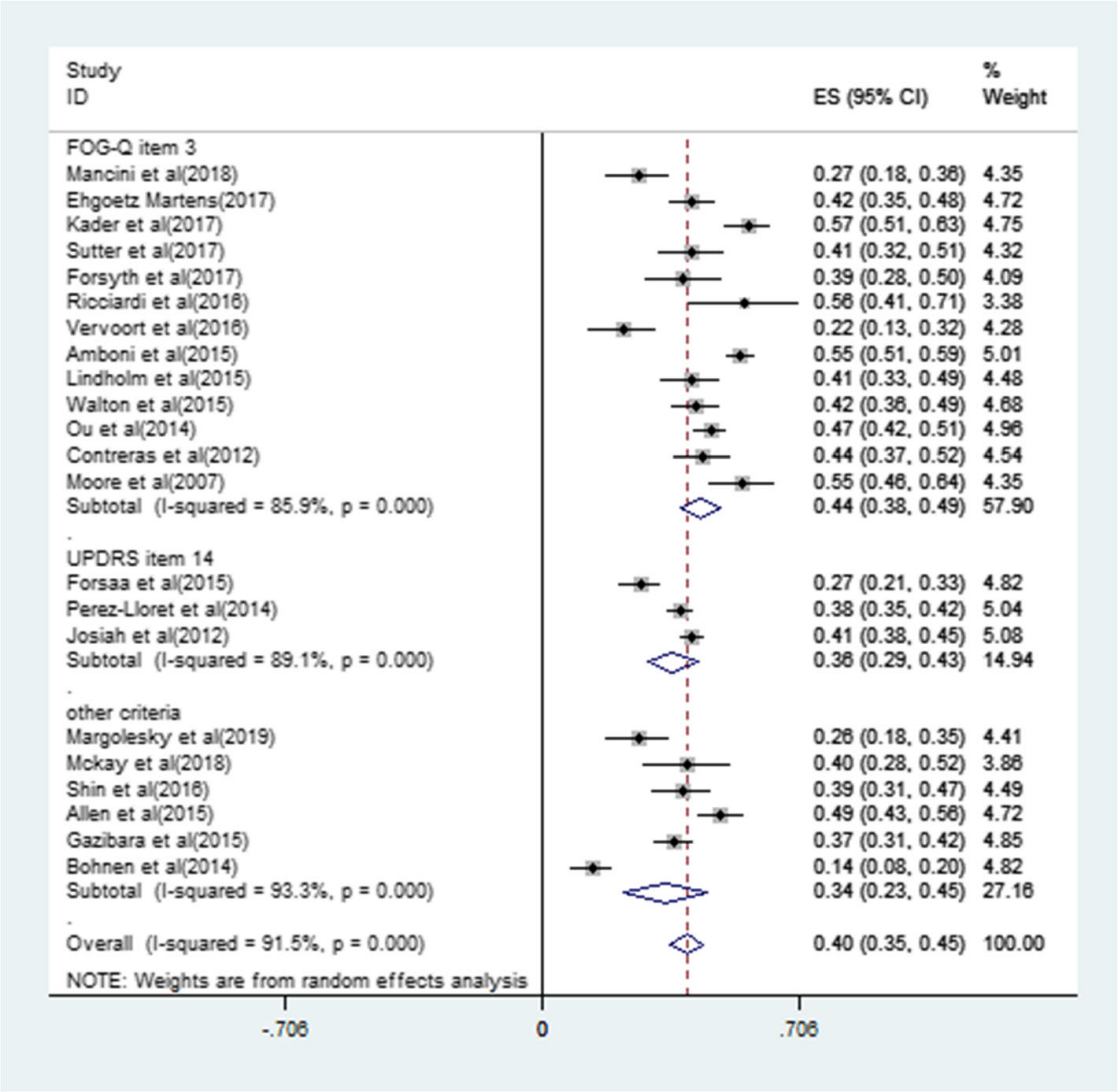
The prevalence of FOG identified by objectively diagnostic criteria

Overall, 39.9% (95%CI 35.3-44.5%) in a total of 5361 PD patients experienced FOG. The FOG identified by the FOG-Q item 3 might be more prevalent (43.8%, 95%CI 38.5-49.1%) than FOG identified by the UPDRS item 14 (36.0%, 29.0-43.1%) (Table 4, Fig. 2). In addition, the prevalence of FOG identified by objectively diagnostic criteria did not have a statistical difference with subjectively diagnosed one (40.2%, 95% CI 35.1-45.2% vs 38.4%, 95% CI 25.2-51.6%, *P* = 0.442) (Table 4).

**Table 4:** The weighted prevalence of FOG in Parkinson’s disease patients and the heterogeneities

Prevalence of FOG	Number of studies	Total no. PD patients	Range of the prevalence of FOG, %	Mean prevalence of FOG (95% CI) ,%	Heterogeneity, % (*P* value)
In general PD patients	22	5361	14.0-56.8	39.9 (35.3-44.5)	91.5% (< 0.001)
According to FOG-Q item 3	13	2559	22.4-56.8	43.8 (38.5-49.1)	85.9% (< 0.001)
According to UPDRS item 14	3	1820	27.2-41.5	36.0 (29.0-43.1)	89.1% (< 0.001)
According to other criteria	6	982	14.0-49.4	34.1 (22.8-45.4)	93.3% (< 0.001)
In PD patients with objectively or subjectively diagnostic criteria					
Objectively	19	4887	14.0-56.8	40.2(35.1-45.2)	92.2% (< 0.001)
Subjectively	3	474	26.5-49.4	38.4(25.2-51.6)	88.8% (< 0.001)
In PD patients with different disease durations					
< 5 years	6	1139	4.4-36.1	22.4 (12.8-31.9)	93.9% (< 0.001)
≥ 5 years	7	1246	34.8-65.8	53.3 (45.8-60.8)	84.1% (< 0.001)
≥ 10 years	6	709	59.1-86.5	70.8 (60.8-80.9)	88.2% (< 0.001)
In PD patients with different H&Y stagings					
≤ 2.5	7	2229	7.1-37.0	28.4 (15.7-41.1)	97.9% (< 0.001)
> 2.5	7	829	60.0-82.9	68.4 (58.7-78.2)	88.4% (< 0.001)

*FOG-Q* freezing of gait questionnaire, *UPDRS* unified Parkinson’s disease rating scale, *FOG* freezing of gait, *PD* Parkinson’s disease, *H&Y* Hoehn and Yahr staging

For the prevalence rates of FOG in PD patients with different disease durations (0-5 years, ≥ 5 years, or ≥ 10 years), FOG is most frequently seen in patients with disease duration ≥ 10 years, at a prevalence rate of 70.8%, followed by patients with disease duration ≥ 5 years (53.3%), and in patients with disease duration < 5 years (22.4%).

In terms of the level of severity, FOG presented in 28.4% of PD patients with H&Y score ≤ 2.5, and in 68.4% of PD patients with H&Y score > 2.5 (Table 4).

Prevalence in recent studies (2016-2019, *n* = 11) was similar to older studies (2007-2015, *n* = 11). In addition, the prevalence in different regions was similar to each other (Table 5).

**Table 5:** Sub-group analysis of the FOG prevalence in general PD patients by the quality of included studies, date of study conduction, and geographical region

	Number of studies	Prevalence	95% CI	*P* value	Heterogeneity, %
Quality score					
14-15	8	40.0%	31.8-48.2%	< 0.001	92.5%
16-17	10	39.7%	32.6-46.8%	< 0.001	90.1%
18-19	4	40.1%	28.0-52.3%	< 0.001	94.5%
Geographic region					
Europe	9	41.7%	34.1-49.4%	< 0.001	93.0%
Asia	3	42.9%	28.9-56.8%	< 0.001	90.7%
United States	6	33.5%	22.8-44.2%	< 0.001	93.4%
Australia	4	43.7%	39.4-48.1%	0.232	30.1%
Year of study conduction					
2007-2015	11	40.1%	33.6-46.6%	< 0.001	94.3%
2016-2019	11	39.7%	32.9-46.6%	< 0.001	86.0%

*FOG* freezing of gait, *PD* Parkinson’s disease

### Heterogeneity

High heterogeneity was found among the synthesis results of included studies. Therefore, we performed subgroup analysis of the FOG prevalence in PD patients based on the scale used to diagnose the FOG, disease duration of PD, disease severity of PD, quality of included studies, date of study conducted, and geographical region. Nonetheless, the heterogeneity in almost all subgroups was considerable (Table 4, Table 5).

## Discussion

### Reported prevalence and variation

In this review, the prevalence of FOG in PD is reviewed for the first time. The results of this meta-analysis show that the overall prevalence of FOG in PD is 39.9%. FOG is most frequently seen in PD patients with disease duration ≥ 10 years at 70.8% (60.8-80.9%), followed by PD patients with disease duration ≥ 5 years (53.3%, 95% CI 45.8-60.8%), and PD patients with disease duration < 5 years (22.4%, 95%CI 12.8-31.9%). In terms of severities, FOG was present in 28.4% (95% CI 15.7-41.1%) of PD patients with H&Y score ≤ 2.5, and in 68.4% (95% CI 58.7-78.2%) of PD patients with H&Y score > 2.5. Our results indicate that the prevalence of FOG in PD patients is considerable.

The prevalence of FOG identified by objectively diagnostic criteria did not have a statistical difference with subjectively diagnosed one (40.2%, 95% CI 35.1-45.2% vs 38.4%, 95% CI 25.2-51.6%, *p* = 0.442). However, the FOG identified by the FOG-Q item 3 (43.8%, 95% CI 38.5-49.1%) may be more prevalent than the FOG identified by the UPDRS item 14 (36.0%, 29.0-43.1%). The item 3 of the FOG-Q, in comparison with item 14 of the UPDRS, identifies more PD patients as “freezers,” which indicates that some patients might not report to have experienced FOG when evaluated with UPDRS item 14, but report to have experienced FOG when they are provided with a detailed explanation of what FOG is (FOG-Q item 3) [47].

In this meta-analysis, it is confirmed that the FOG in PD is associated with increased severity (H&Y) and longer disease duration. The disease severity (H&Y) and disease duration had been proven to be strong clinical features associated with FOG [11, 27, 28, 48]. In addition, the H&Y and disease duration may be attributed to the severity of FOG [49–51].

There is a wide range of the FOG prevalence reported in the included studies. The heterogeneity among the results of the included studies was not negligible. These studies were conducted in different regions, different diagnostic criteria of FOG were used, and patients with different durations and severities were included. These differences may be the sources of heterogeneity. However, Chi^2^ test was used in this study, which is highly effective in identifying small heterogeneities that might not be practically important [52]. In the sub-analysis, based on the scale used to diagnose FOG, disease duration, severity of PD, quality of included studies, date of study conducted, and geographical region, the heterogeneity was high. Furthermore, the dissimilarities of the medication states of FOG also contributed to the heterogeneity. The FOG prevalence rates vary in PD patients who experienced FOG only during off state, only during on state, and either in on or off states and is independent of dopaminergic response-related symptoms [11, 53]. Nonetheless, if the results of the meta-analysis are to be used as a recommendation for medical decision-making, it is possible to analyze the heterogeneous results of studies [54]. Furthermore, in order to reduce the heterogeneity, the studies without a sufficient description of a random or consecutive design of patient recruitment have already been excluded, and the random-effects model has been used in this meta-analysis to estimate the prevalence of FOG.

### Inspiration to surgical work

Patients with advanced PD often show the axial symptoms, including gait disorders and postural abnormalities [55]. Gait disorders often occur in the situation when the patients’ attention shifts or the walking direction changes. At that moment, patients often fall down because the movement of legs lags behind the upper body [56], which has a significant adverse effect on the patients’ quality of life [26]. However, the current main treatments of PD patients sometimes do not work in the axial symptoms, including dopamine drug therapy and deep brain stimulation in classical nuclei ( such as subthalamic nucleus and globus pallidus internus) [57]. The pedunculopontine nucleus (PPN) is an important nucleus in the neural activity that controls the initiation of gait. Therefore, it has gained wide attention whether the PPN-DBS could improve the axial symptoms of PD patients [58]. Results in the PD animal model showed that low-frequency PPN-DBS can improve the gait disorders and postural abnormalities [59–61]. More than that, clinical studies have also shown that low-frequency PPN-DBS can selectively improve gait disorders in PD patients [62–64]. In patients with severe freezing of gait and postural abnormalities, which have treated with drugs but not with subthalamic nucleus deep brain stimulation (STN-DBS), deep brain stimulation in the lower and middle parts of the bilateral PPN has shown a good therapeutic effect [65].

In this meta-analysis, we concluded that the overall incidence of freezing of gait was 35.8%. These patients may be suitable for the multi-target DBS because of the insignificant therapeutic effect by dopamine drug therapy and deep brain stimulation in classical nuclei. The therapeutic effect of STN-DBS in the axial symptoms in PD patients remains controversial [66–69]. Some studies suggested that STN-DBS could improve both limb dyskinesia and gait disorders in PD patients [70]. Hamani et al [71] reported that the improvement rate of gait disorders was 64% at 1 year after STN-DBS. Bejjani et al [72] claimed that gait disorders and postural abnormalities improved at 6 months after STN-DBS. STN-DBS may improve gait disorders and postural abnormalities through the direct stimulation on PPN [70, 73]. In addition, Khan et al [74] reported that PPN-DBS combined with STN-DBS improved the axial symptoms in patients with advanced PD. Although multi-target DBS is still in the clinical exploration stage, it is worthwhile to study how to improve multiple symptoms by stimulating multiple nucleus [75]. Our study showed that the overall incidence of FOG in PD patients was high and was related to the disease duration and severity. Therefore, for PD patients, a detailed assessment of FOG is important for formulating a treatment plan and could not be ignored.

### Limitations

There are a number of limitations in this meta-analysis. First, despite the attempts to identify all suitable publications and to exclude publications stemming from the same database, it is possible that some of the overlapping publications might be missed. Second, although quality criteria were used in all of the papers studied, the inclusion criteria used were subjective to the authors. This may increase the possibility of information bias. Third, the medication state of FOG in most studies was unknown. Therefore, a sub-analysis of the FOG prevalent based on the medication state cannot be performed. Lastly, the possible influences of pharmacological treatment of FOG cannot be ruled out For example, patients who had FOG but were successfully treated may not be recognized, and this may lead to an underreporting of FOG.

However, taking all of these considerations into account, the overall prevalence rate of FOG in available studies was calculated with the intention to provide a reliable estimation of the rate of FOG in PD patients. Although FOG in PD was relatively neglected for a long time, it has received more attention in the past decade. By demonstrating a high prevalence of FOG in PD (39.9%), the present meta-analysis highlights the need for accurate identification of FOG in PD.

## Conclusions

This is the first review on the prevalence of FOG in PD. The results showed that the prevalence of FOG in PD is 35.8% and it varied with different disease durations and severities. It reminded us that physicians should be aware of FOG as a common feature in PD. More accurate diagnostic rating scale and efficient treatments such as multi-target DBS should be further studied and optimized for PD patients to increase their quality of life.

## Data Availability

All data generated or analyzed during this study are included in the article.

## Abbreviations

FOG: Freezing of gait
PD: Parkinson’s disease
H&Y: Hoehn and Yahr
QUADAS: Assessment of diagnostic accuracy studies
Chi2: Chi-square
FOG-Q: Freezing of gait questionnaire
UPDRS: Unified Parkinson’s Disease Rating Scale
PPN: Pedunculopontine nucleus
STN: Subthalamic nucleus
